# Association of early initiation of breastfeeding on postpartum depression—multi-centric longitudinal cohort study in Nepal

**DOI:** 10.3389/fgwh.2026.1752660

**Published:** 2026-05-15

**Authors:** Ashish KC, Ankit Acharya, Omkar Basnet, Luong Nguyen Thanh, Maria Karalexi, Maria Grandahl, Honey Malla, Rejina Gurung, Alkistis Skalkidou

**Affiliations:** 1 Institute of Medicine, University of Gothenburg, Gothenburg, Sweden; 2 Department of Women’s and Children’s Health, Uppsala University, Uppsala, Sweden; 3 Research Division, Golden Community, Lalitpur, Nepal; 4 Department of Pediatrics, Hellenic Society for Social Pediatrics and Health Promotion, Athens, Greece

**Keywords:** early breast feeding, longitudinal cohort, Nepal, postpartum depression, socially disadvantaged

## Abstract

**Background:**

Evidence of the relationship between breastfeeding and maternal mental health is mixed and complex, with some studies suggesting breastfeeding may lower the risk for postpartum depressive symptoms, while others report no clear or consistent effects. Given these inconsistencies, we aim to assess the association between the timing of initiation of breastfeeding and postpartum depressive symptoms 90 days after birth in Nepal.

**Methodology:**

This longitudinal multi-centric cohort study included 898 mother-infant pairs in 9 district hospitals of Nepal. Data was collected on timing of initiation of breastfeeding, sociodemographic variables and depressive symptoms assessed through the Edinburg Postnatal Depression Scale. A Directed Acyclic Graph was constructed and multiple logistic regression, generalized mixed linear regression model and Generalized Estimating Equations (GEE) were used to assess the association of timing of breastfeeding with postpartum depressive symptoms.

**Principal results:**

At the 90th day postpartum, 31.4% of women reported depressive symptoms. Compared to women who had immediate breastfeeding, those who had no immediate breastfeeding had 3.47 higher odds of depressive symptoms (cOR: 3.47; 95% CI; 2.40, 5.01). After adjusting for confounding and mediating factors, the odds of depressive symptoms were 2.81 times higher among women who did not immediately breastfeed (aOR, 2.81; 95% CI; 1.76, 4.50). Using GEE modeling, there was a positive association between delayed breastfeeding and postpartum depression at 7 days (β coefficient, 0.583, *p* = 0.001) and at 45 days (β coefficient, 0.551, *p* = 0.003). Using the generalized linear mixed model, the prediction to postpartum depression score increased with delay in breastfeeding.

**Conclusions:**

This study highlights that the delayed initiation of breastfeeding is associated with higher odds of symptoms for postpartum depression among various groups of women, especially among women from disadvantageous groups and women with no education in Nepal. Improving support to women for early initiation of breastfeeding could help reduce postpartum depression.

## Introduction

Globally, of the 140 million women who give birth annually, 40% initiate early breastfeeding (within 1 h of childbirth) (1). Although evidence suggests that early initiation of breastfeeding reduces the risk of neonatal infections, infant mortality and enhances optimal child's development, the rate of early initiation of breastfeeding has not improved significantly (2). Disruption of this normal oxytocin (OT) physiology has been linked to dysregulated stress response and poor feeding outcomes (3, 4). Abnormalities in OT signaling and dysregulation of the hypothalamic pituitary adrenal (HPA) stress axis, including glucocorticoid signaling and impaired feedback regulation of the relevant stress response system, have been shown in women with postpartum depression (5, 6). Postpartum depression is a rising global health problem with an increasing annual rate of around 15%–20% (7). Furthermore, postpartum mental health status is of particular concern as women undergo a surge of family and social adaption (7).

The perinatal health system in Nepal faces considerable challenges that shape maternal and neonatal outcomes. Despite several national level initiatives for perinatal care (8), only five out of twelve hospitals had all basic newborn care services, Kangaroo-Mother-Care was absent in most hospitals, and just 8.2% of mothers-initiated breastfeeding before transferring to the postnatal ward (9). These infrastructural and resource limitations, combined with sociocultural factors, create a different context from high-income countries. A large-scale study in Nepal found that more than two third of newborns were not breastfed within one hour after birth, and only 3.5% of newborns were kept in skin-to-skin contact with their mothers (10). In Nepal, one fourth of women had depressive symptoms postnatally which further impairs children's growth and development (11). Postpartum depression can directly and indirectly alter an infant's brain and neurocognitive development. It has been identified that postpartum depression can have a significant effect on the structure and function of children's pre-frontal cortex, as well as their intrauterine cognitive development abilities (12). A systematic review study showed that postpartum depression contributes to an environment which is not suitable for the optimal development of a child (13).

Multiple social risk factors are associated with postpartum depression such as ethnicity, child's sex and other sociodemographic factors (14). Evidence also indicates that breastfeeding practices, particularly early initiation and sustained duration, may play an important role. A systematic review found a protective association between breastfeeding and maternal mental health, as seen in fewer symptoms of postpartum depression and anxiety (15). Another study found that delayed initiation and shorter duration of breastfeeding were linked to higher rates of postpartum depression (16). However, no study has explored this association in the Nepali context, where unique socio-cultural factors shape healthcare factors and practices. In this multi-centric cohort study, we aim to assess the association of early initiation of breastfeeding with postpartum depression at 90 days after childbirth in a cohort of mother-infant pairs in Nepal.

## Materials and methods

### Research design

This is a multi-centric, prospective cohort study conducted among women who gave birth in nine hospitals between May and August 2020, representing the general population of women delivering in these facilities. Women were followed up before childbirth until 90 days after birth. Ethical approval was obtained from Nepal Health Research Council (17, 18).

### Setting and relevant context

Almost two third of the women delivered in health facilities of Nepal (19). The selected nine hospitals were the referral centers representing all seven provinces of the country. Each hospital had human resources and infrastructure for managing complicated deliveries using instrumental or cesarean section.

### Inclusion criteria

Participants giving childbirth in labour unit were eligible for the study. Eligible women were informed, and written consent was obtained from all participants. Women who had liveborn neonates were included for clinical observation and cohort follow-up. Women delivered using cesarean section, who had stillbirth or delivered a newborn with a gestational age below 28 weeks, or gave birth to neonates with congenital malformations were excluded.

### Sample

**Figure 1:**
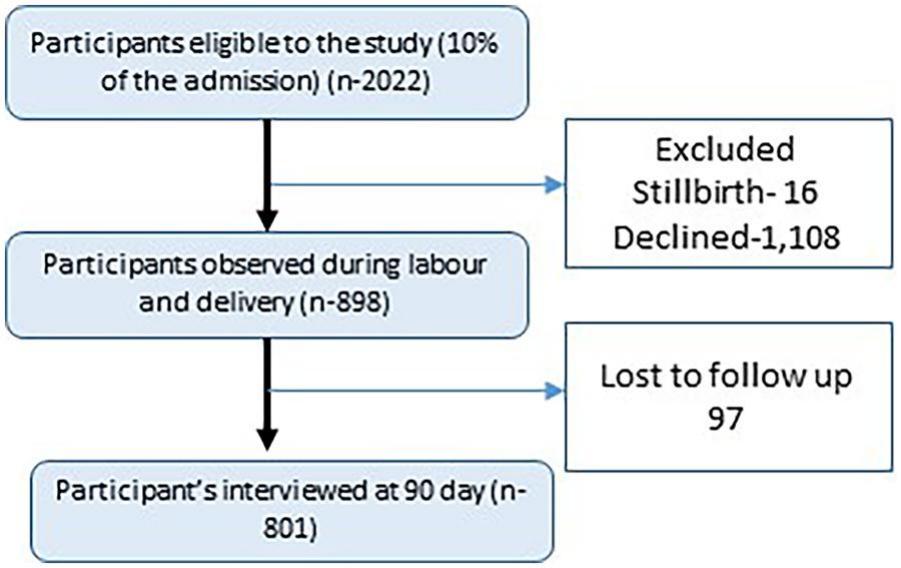
Study flow figure.

We conducted this study as part of a multi-centric prospective cohort study examining maternal and neonatal health outcomes in nine hospitals in Nepal. During the study period, 21,805 women met the eligibility criteria for enrollment. From this population, 10% (*n* = 2022) were randomly selected using computer-generated random sequence generation in Microsoft Excel for potential inclusion in the longitudinal follow up. Among the 2022 women selected for the study, clinical observation of immediate newborn care (including breast feeding) in delivery room was available for 898 mother-newborn pairs. The remaining women were not observed due to the limited availability of data surveillance personnel to observe all participants during birth and immediate postnatal period. Of the 898 mother-newborn pair who were observed for immediate newborn care, 801 completed the follow up interview at 90 days postpartum and were included in the final analytical sample assessing postpartum depressive symptoms (Figure 1). The depression symptoms were assessed using the Edinburgh Postpartum Depression Scale (EPDS) (20, 21).

Sample size: We calculated the sample size based on the expected prevalence of postpartum depressive symptoms in Nepal. A previous study in the country reported prevalence of approximately 21% (22) and assuming a prevalence of delayed initiation of breast feeding of approximately 31%, a two-sided alpha level of 0.05% and 80% statistical power, a sample of approximately 900 participants was estimated to detect association between delayed initiation of breast feeding and postpartum depression. However, the cohort was not specifically designed to evaluate early initiation of breast feeding as the primary exposure to postpartum depression and therefore this is an exploratory analysis.

## Measurement

### Outcome variable

The EPDS compiled by Cox et al. (21) was used in this study for assessing postpartum depression. It includes ten items; each scored between zero and three by severity on a four-point scale. The EPDS has shown good reliability and validity and can be used to assess depression during pregnancy and after delivery. Although the tool had previously been validated in Kathmandu, Nepal (20), the research team conducted an additional validation to account for the different socio-economic settings across all provinces of the country. The items were first translated into the local Nepali language, and then back-translated into English by independent translators. In this study, women with a total score of nine or higher were categorized as having depressive symptoms. A lower cut-off score was used since both the seminal work of Cox JL on the validation of the EPDS and studies from Nepal showed that using a cut-off score of nine or ten reduces failed case detection to less than 10% (20, 23).

### Exposure

The exposure variable was timing to first breastfeeding in the delivery room, which was assessed by the data surveillance team through observation. The timing to first breastfeeding in delivery room was recorded in minutes. In this study, immediate breastfeeding refers to breastfeeding initiated within one hour after birth. If breastfeeding was started after one hour, it was considered as delayed initiation of breastfeeding.

### Other variables

*Sense of Coherence (SOC)* was assessed at 90 days, SOC-13 (24), which measures mental resilience during postpartum period. The cut-off score for SOC was 60 i.e., a score of less than 60 was categorized as low SOC and 60 or more was categorized as high SOC. *Caste-* The social hierarchy system predetermining the access to social resources, for this study purpose it was divided into two groups, relatively disadvantaged groups which included Janjati, Muslim, Madeshi and Dalit caste group and relatively advantaged groups such as Chettri-Brahmin. *Maternal age-* age categories were 18 years or less, 19–24 years, 25–29 years, 30–34 years, and over 35 years old. *Maternal education* was classified into two categories: not educated (illiterate and not able to read and write) and educated, including those who had primary, secondary or higher secondary level of education. *Parity-* Parity was categorized as nulliparous (women who had no previous births), primiparous (women with at least one previous birth) and multiparous (women having two or more previous births). *Mode of childbirth-* Categorized as spontaneous vaginal and assisted vaginal birth. *Gestational age-* Using the last menstrual cycle count; preterm birth defined as birth prior to 37 weeks of gestation. Although early-pregnancy ultrasound is considered the gold-standard for gestational age estimation, it was not consistently available across all study hospitals. *Birth weight-* Birth weight classified as birth weight <2,500 grams and birth weight 2,500 grams or more. *Sex-* Sex of the newborn as either male or female.

### Data collection

A trained data surveillance team on the data collection was set up in each hospital. The data surveillance team informed the eligible women about the study and enrolled those who provided written consent. An information sheet was provided to the enrolled woman on the purpose of the study and the follow up of the woman during the postpartum period. The surveillance team collected the participant's socio-demographic, obstetric and immediate newborn information by interviewing the women at the time of discharge.

The telephone follow-up interview was conducted by the surveillance team at 90 days after childbirth (Figure 1). All data was collected using a tablet-based application, which was validated in a large-scale study on quality improvement (18).

### Data management and analysis

**Figure 2:**
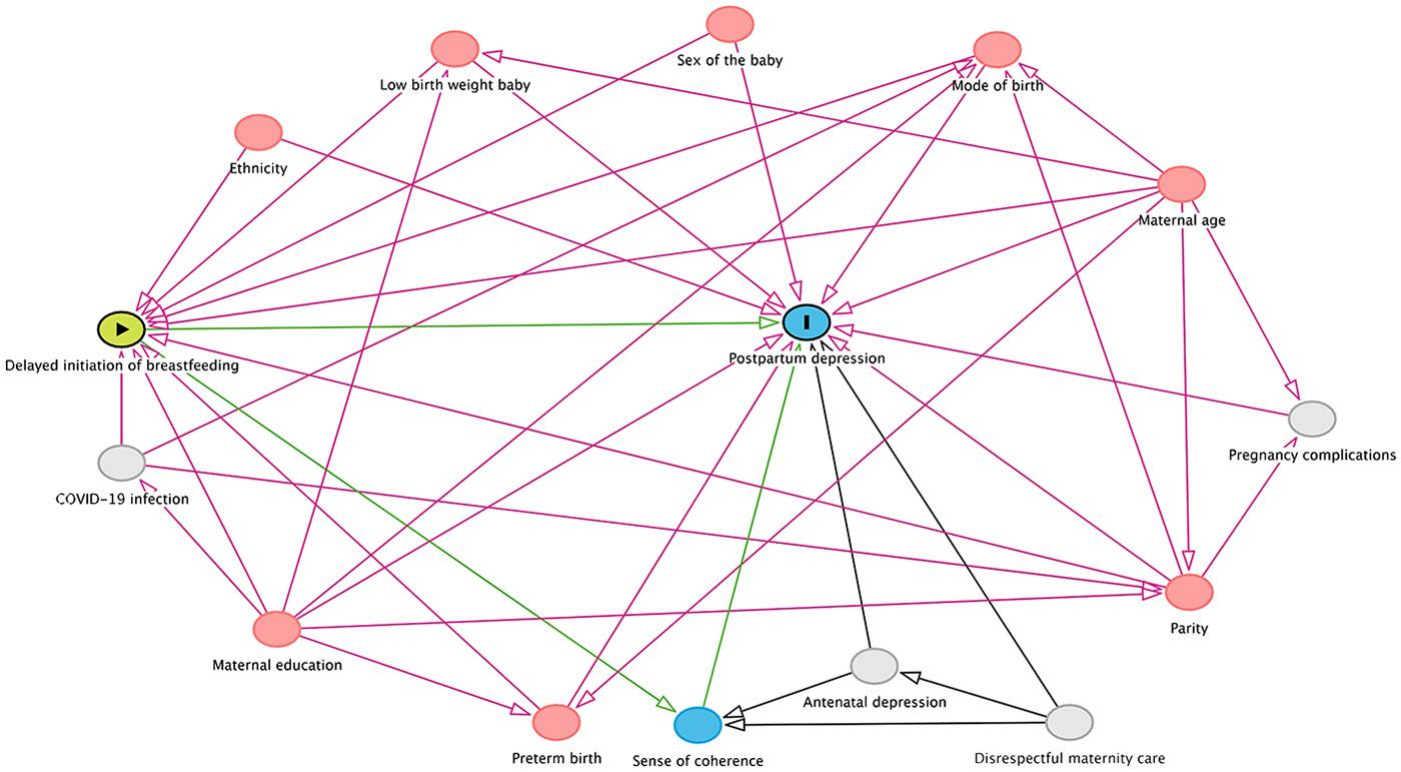
Directed acyclic graph (DAG) showing the relationship between delayed breastfeeding initiation and depression in postpartum women. *The main exposure and the outcome are shown by the green and blue nodes with symbols inside. The red nodes represent adjusted confounders, whereas the blue node represents a mediator that is a part of the causal pathways (green arrows) between delayed breastfeeding initiation and postpartum depression. Unobserved (latent) variables are shown by grey nodes*.

Based on literature review, we developed a conceptual framework using the directed acyclic graph (DAG) to identify potential confounders and mediators in the relationship between initiation of breastfeeding and postpartum depression (25). Based on the DAG methodology, maternal education, maternal age, ethnicity, parity, sex of the baby, mode of birth, preterm status, were considered as confounding variables; immediate breastfeeding was defined as the exposure variable; and postpartum depression was defined as the outcome variable. SOC in this model acts as potential mediator, since it is affected by the exposure variables and affects the outcome variable (Figure 2).

Descriptive statistics were used to summarize maternal socio-demographic, obstetric and neonatal characteristics in the exposure and outcome group. Since the prevalence of postpartum depressive symptoms in this cohort was relatively high, we conducted odds ratios from logistic regression rather than relative risk using other methods to assess the association between exposure variable and outcome variable. Bivariate logistic regressions were performed to assess association between immediate breastfeeding and postpartum depression. Multi-variable logistic regression was conducted to assess the association of immediate breastfeeding with postpartum depression (Figure 2). Three models were constructed based on the conceptual framework. Model I included unadjusted odds ratio; model II- factors impacting on both exposure and outcome (confounders) and model III- factors impacting on both exposure and outcome, or those impacting only on outcome (some are a result of the exposure and thus can act as mediators).

Generalized Estimating Equation (GEE) was used to assess the strength of association between early initiation of breastfeeding with postpartum depression score. A generalized linear mixed model was employed to assess the timing of breastfeeding with postpartum depression score. Statistical analysis was conducted using IBM SPSS Statistics Version 26 and R-studio.

## Results

A total of 2022 women were randomly selected from eligible deliveries from the participating hospitals. Among these, clinical observation was available for 898 mother-infant pairs and 801 were followed up until day 90 after childbirth (Figure 1). Comparisons between the initially sampled population (*n* = 2,022) and the observed cohort (*n* = 898) indicated statistically significant differences in several socio-demographic and obstetric characteristics, including maternal education, ethnicity, parity, preterm birth and birth weight (Supplementary Table S1).

All of the mothers observed (*n* = 801) were breastfeeding at some point after birth. 412 women (51.4%) had early initiation of breastfeeding (within one hour) with the median time of 31 min (IQR 20–46). Women from socially disadvantaged families have lower proportion of early initiation of breastfeeding than those from socially disadvantaged family (47.3% vs. 61.2%, *p*-value < 0.001). Women who have premature birth have higher proportion of early initiation of breastfeeding then those who have term birth (78.4% vs. 50.1%, *p*-value = 0.002). Among mothers who immediately breastfed, the SOC >60 was higher compared to mothers who did not immediately breastfeed (57.9% vs. 42.1%, *p*-value = 0.042) (Table 1).

**Table 1:** Demographics for early initiation and delayed initiation of breastfeeding.

Indicators/Variables	Early initiation of breast feeding (*n* = 412)	Delayed initiation of breast feeding (*n* = 389)	*p*-value
Maternal education*			
Educated (538)	293 (54.5%)	245 (45.5%)	Reference
Not educated (89)	32 (36.0%)	57 (64.0%)	0.001
Ethnicity			
Relatively advantaged (237)	145 (61.2%)	92 (38.8%)	Reference
Relatively disadvantaged (564)	267 (47.3%)	297 (52.7%)	<0.001
Maternal age			
Less than 18 (33)	15 (45.5%)	18 (54.5%)	0.449
19–24 years (409)	214 (52.3%)	195 (47.7%)	Reference
25–29 years (258)	128 (49.6%)	130 (50.4%)	0.495
30–34 years (77)	37 (48.1%)	40 (51.9%)	0.492
35 year or more (24)	18 (75.0%)	6 (25.0%)	0.037
Parity			
No previous birth (275)	158 (57.5%)	117 (42.5%)	0.007
One previous birth (323)	150 (46.4%)	173 (53.6%)	Reference
More than one previous birth (203)	104 (51.2%)	99 (48.8%)	0.285
Mode of birth			
Assisted birth (36)	13 (36.1%)	23 (63.9%)	Reference
Spontaneous Vaginal (765)	399 (52.2%)	366 (47.8%)	0.064
Preterm			
No (764)	383 (50.1%)	381 (49.9%)	Reference
Yes (37)	29 (78.4%)	8 (21.6%)	0.002
Low Birth Weight			
No (638)	341 (53.4%)	297 (46.6%)	Reference
Yes (163)	71 (43.6%)	92 (56.4%)	0.025
Infant's Sex			
Boy (443)	228 (51.5%)	215 (48.5%)	Reference
Girl (358)	184 (51.4%)	174 (48.6%)	0.984
SOC at 90 days			
60–74 (190)	110 (57.9%)	80 (42.1%)	Reference
<60 (611)	302 (49.4%)	309 (50.6%)	0.042

* Missing = 174.

The prevalence of having depressive symptoms among followed 801 participants was 21.2% with 31.4% of women who had no immediate breastfeeding reporting depressive symptoms. After adjusting for confounding factors, the odds were 2.99 times higher among those who did not immediately breastfeed (aOR, 2.99; 95% CI; 1.89, 4.73). After adjusting for confounding and mediating factors, the odds of depressive symptoms were 2.81 times higher among women who did not immediately breastfeed (aOR, 2.81; 95% CI; 1.76, 4.50) (Table 2).

**Table 2:** Association between immediate breastfeeding and postpartum depression symptoms at 90 days after birth, adjusted also for relevant socio-demographic, obstetric and neonatal characteristics.

Indicators/Variables	No depressive symptom (631)	Depressive symptom (170)	Model I; cOR, 95% CI†	Model II; aOR 95% CI‡	Model III; aOR 95% CI¥
Immediate breastfeeding					
Yes (412)	364 (88.3%)	48 (11.7%)	Reference	Reference	Reference
No (389)	267 (68.6%)	122 (31.4%)	3.47 (2.40, 5.01)	2.99 (1.89, 4.73)	2.81 (1.76, 4.50)
Maternal education*					
Educated (538)	447 (83.1%)	91 (16.9%)	Reference	Reference	Reference
Uneducated (89)	34 (38.2%)	55 (61.8%)	7.95 (4.90, 12.89)	4.01 (2.28, 7.05)	4.06 (2.27, 7.27)
Ethnicity					
Relatively advantaged (237)	216 (91.1%)	21 (8.9%)	Reference	Reference	Reference
Relatively disadvantaged (564)	415 (73.6%)	149 (26.4%)	3.69 (2.27, 6.00)	2.76 (1.50, 5.09)	2.65 (1.42, 4.95)
Maternal age					
Less than 18 (33)	24 (72.7%)	9 (27.3%)	1.62 (0.72, 3.62)	1.71 (0.55, 5.26)	1.57 (0.50, 4.92)
19–24 years (409)	332 (81.2%)	77 (18.8%)	Reference	Reference	Reference
25–29 years (258)	203 (78.7%)	55 (21.3%)	1.17 (0.79, 1.72)	0.94 (0.56, 1.56)	1.04 (0.62, 1.76)
30–34 years (77)	52 (67.5%)	25 (32.5%)	2.07 (1.21, 3.55)	0.79 (0.37, 1.65)	0.83 (0.39, 1.79)
35 year or more (24)	20 (83.3%)	4 (16.7%)	0.86 (0.29, 2.60)	0.33 (0.09, 1.17)	0.32 (0.09, 1.15)
Parity					
No previous birth (275)	255 (92.7%)	20 (7.3%)	0.34 (0.20, 0.57)	0.33 (0.17, 0.64)	0.42 (0.21, 0.81)
1 previous birth (323)	262 (81.1%)	61 (18.9%)	Reference	Reference	Reference
≥2 previous birth (203)	114 (56.2%)	89 (43.8%)	3.35 (2.26, 4.97)	3.26 (1.96, 5.43)	3.03 (1.80, 5.10)
Mode of birth					
Spontaneous vaginal birth (765)	602 (78.7%)	163 (21.3%)	Reference		
Assisted Vaginal (36)	29 (80.6%)	7 (19.4%)	0.89 (0.38, 2.07)		
Preterm					
No (764)	599 (78.4%)	165 (21.6%)	Reference		
Yes (37)	32 (86.5%)	5 (13.5%)	0.57 (0.22, 1.48)		
Low Birth Weight					
No (638)	494 (77.4%)	144 (22.6%)	Reference		
Yes (163)	137 (84.0%)	26 (16.0%)	0.65 (0.41, 1.03)		
Infant's sex					
Girl (358)	294 (82.1%)	64 (17.9%)	0.69 (0.49, 0.98)	0.75 (0.49, 1.18)	0.68 (0.43, 1.08)
Boy (443)	337 (76.1%)	106 (23.9%)	Reference	Reference	Reference
SOC at 90 days					
60–74 score (190)	184 (96.8%)	6 (3.2%)	Reference		Reference
Less than 60 score (611)	447 (73.2%)	164 (26.8%)	11.25 (4.89, 25.89)		4.83 (2.22, 10.50)

*Missing values.

† Model I, unadjusted odds ratio.

‡ Model II, confounding factors associated with depressive symptom.

¥ Model III, confounding and mediating factors associated with depressive symptom.

**Figure 3:**
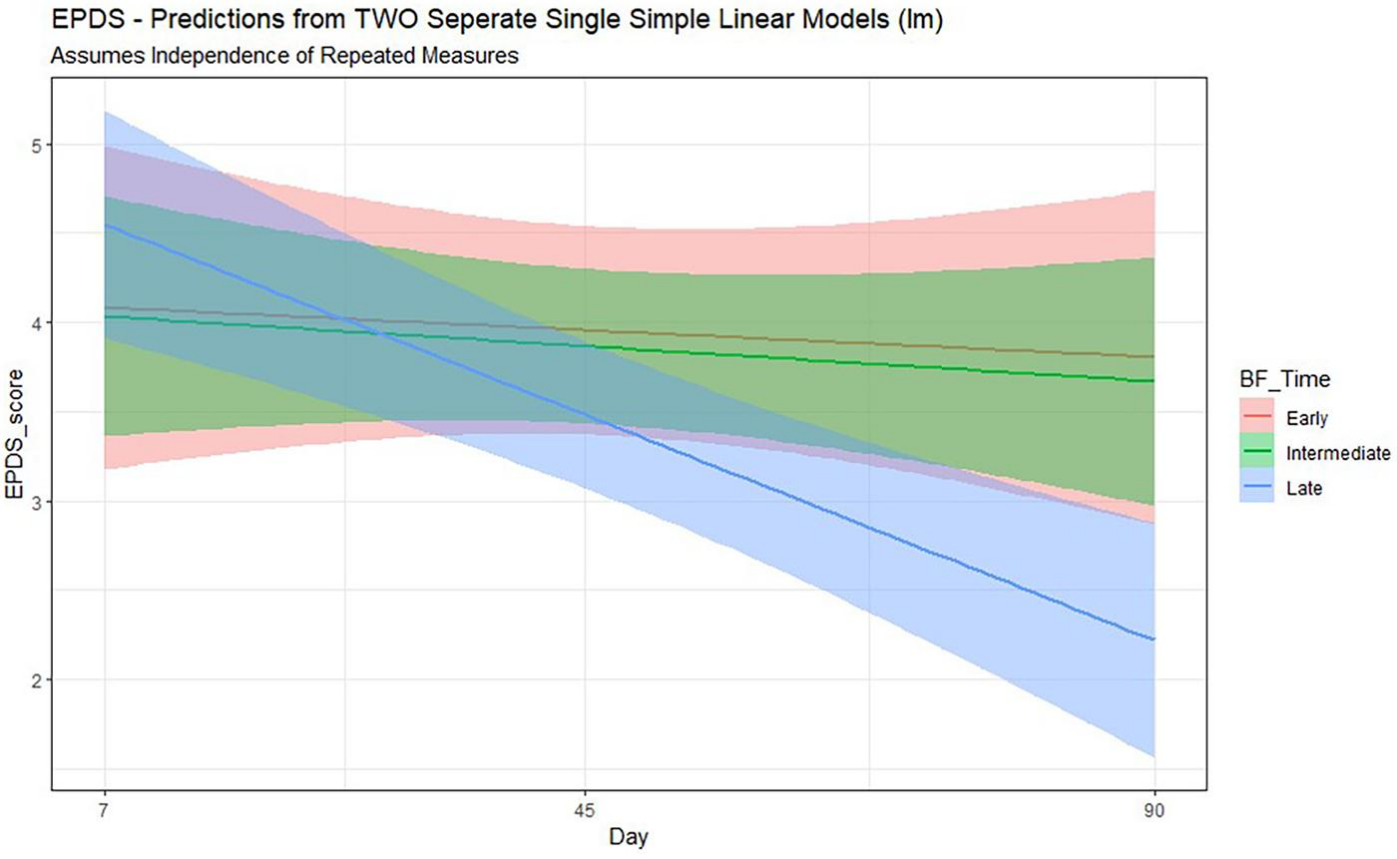
Timing of breastfeeding and prediction of EPDS score on 90th day.

Using the GEE modeling, delayed breastfeeding was significantly and positively associated with postpartum depression (coeff. 1.06, *p* < 0.001), after adjusting for confounding and mediating factors. Women who are uneducated and are from socially disadvantaged ethnicity with low SOC have increased possibility of depression (Table 3). Using the generalized linear mixed model, after adjusting for confounding and mediating factors, the prediction to postpartum depression score increased with delay in breastfeeding (Figure 3).

**Table 3:** Results of the generalized estimating equations of postpartum depressive symptoms with associated factors (*n* = 801).

	Model I†		Model II†	
	B-coefficient	*p*-value	B-coefficient	*p*-value
*Intercept*	−2.837	<0.001	−4.854	<0.001
Immediate breastfeeding				
Yes	Reference		Reference	
No	1.093	<0.001	1.057	<0.001
Maternal education*				
Educated	Reference		Reference	
Uneducated	1.388	<0.001	1.364	<0.001
Ethnicity				
Relatively advantaged	Reference		Reference	
Relatively disadvantaged	1.015	0.002	0.998	0.003
Maternal age				
Less than 18	0.535	0.310	0.184	0.730
19–24 years	Reference		Reference	
25–29 years	−0.064	0.805	−0.208	0.441
30–34 years	−0.242	0.513	−0.376	0.351
35 year or more	−1.113	0.072	−1.259	0.047
Parity				
No previous birth	−1.098	0.002	−1.044	0.004
1 previous birth	Reference		Reference	
2 or more previous birth	1.182	<0.001	1.247	<0.001
Infant's sex				
Girl	−0.288	0.200	−0.384	0.096
Boy	Reference		Reference	
SOC at 90days				
60–74 score			Reference	
Less than 60 score			2.454	<0.001

* Missing 174.

† Unadjusted odds ratios.

## Discussion

In this study, half of the population did not initiate breastfeeding within the first hour of delivery, and delayed initiation of breastfeeding was strongly associated with postpartum depressive symptoms at 90 days, even after controlling for possible co-founders. The onset of depressive symptoms was inversely associated with early breastfeeding. There were multiple social, demographic and obstetric risk factors for women to develop depressive symptoms during postpartum period such as women from relative disadvantaged community, women with no education, women who had more than two children previously and those with low SOC. This finding underscores the need for healthcare providers in Nepal to promote early breastfeeding practices as a crucial strategy for improving maternal mental health, particularly in low-resource settings.

Addressing these risk factors is essential for implementing effective breastfeeding interventions that cater to the unique needs of vulnerable populations in Nepal (26). However, these interventions must also be understood within the broader context of Nepal's perinatal health system. Despite availability of clinical standard protocol for high quality care for mothers and newborns in public hospitals, the adherence to standards has been poor (10). Nevertheless, national initiatives, such as the “Every Newborn Action Plan” and “Breastfeeding Promotion Programs,” have contributed to a steady rise in early breastfeeding rates (19). These programs aim to integrate essential maternal and neonatal health interventions, with a strong focus on early breastfeeding and good practices during childbirth and delivery. Enhanced training for healthcare providers can improve adherence to these protocols and support mothers in initiating breastfeeding promptly. Quality improvement interventions have been piloted in public referral hospitals to strengthen immediate breastfeeding support (18). The Baby-Friendly Hospital Initiative (BFHI), introduced in Nepal in 1994, is an important milestone, however, by 2012 only seven hospitals had been certified and more recent data on scale-up is limited. The sustainability of BFHI practices requires ongoing investment, regular monitoring, and training of health personnel (27). Our recent study showed that initiation of skin-to-skin contact or zero separation at birth is a strong predictor for early initiation of breastfeeding (28). Strengthening provider training to ensure zero separation could be a feasible and impactful intervention for early initiation of breastfeeding and improved neonatal outcomes (29).

Beyond neonatal benefits, early breastfeeding appears to support maternal mental well-being. Women who breastfeed within two hours after delivery reported higher SOC and more positive feelings and satisfaction compared to those who did not breastfeed (30), suggesting that immediate breastfeeding could serve as an effective intervention for improving maternal psychological well-being in the context of Nepal. Similarly, non-exclusive breastfeeding was associated with poor sleep quality and higher depressive symptoms (31). Our study also showed that early breastfeeding had a higher SOC at 90 days, indicating SOC as mediating factor between immediate breastfeeding and postpartum depression. While our study showed an association between postpartum depression and initiation of breastfeeding, the absence of data regarding previous mood disorders of participants may have influenced these findings as pre-existing mood are a strong predictor for postpartum depression (32).

The relationship between breastfeeding and postpartum depression is likely bidirectional. While our study focused on whether early initiation of breastfeeding protects against depressive symptoms, several studies, such as from the UK (33) as well as from South Asia and other LMICs have reported the reverse association—that mothers with perinatal depression are more likely to delay initiation, shorten exclusive breastfeeding duration, or end breastfeeding early (34). Since our study did not exclude women with a history of antepartum depression, these findings are highly relevant for Nepal, as well as due to similarities in health system capacity, sociocultural context, and resource constraints. At the same time, studies from high-income settings (HICs), such as the US have shown that late breastfeeding was associated with postpartum depression and early breastfeeding was protective against late postpartum depression (35, 36). A study in UAE showed that higher scores on EPDS and diagnosis of postpartum depression at two months had higher predictive value of lower rates of breastfeeding at four months. Women who breastfed their infants reduced the risk for postpartum depression, with effects being maintained over the first four months postpartum (37). This suggests that the association between perinatal depression and breastfeeding practices (and vice versa) is consistent across diverse health system contexts. However, the underlying mechanisms may differ; in HICs the challenge may be psychological support and maternal choice, while in Nepal the above-mentioned challenges may play a larger role. These findings indicate that enhancing breastfeeding practices and addressing postpartum mental health are mutually reinforcing goals. Integrating mental health support into maternal health care services in Nepal is therefore critical, including counselling on the benefits of early breastfeeding, strategies to cope with postpartum challenges, and interventions tailored to the cultural and socio-economic context of Nepalese women (38).

Lastly, broader structural factors may also shape maternal mental health and access to early breastfeeding support in Nepal. The country ranks 117th out of 146 countries in the Global Gender Gap Index 2024, with persistent disparities in economic participation and limited female representation in national political leadership (39). For instance, only 27.6% of women participate in the labor force compared to 53.7% of men. Although women are increasingly represented in local governance, their presence in national-level political leadership remains limited. These figures highlight persistent structural inequalities that may influence maternal health outcomes, including access to perinatal care, decision-making power in healthcare, and support for mental health (40).

Methodological consideration—This study has several methodological strengths and limitations that should be considered when interpreting the results. One of the strengths is the prospective cohort design, which provides for causality between exposure to outcome due to temporality and biological gradient. The study was conducted in 9 hospitals of the country, which provides provincial representation on the prevalence of breast-feeding initiation as well as postpartum mental health. Finally, since, breast feeding initiation was directly observed in the delivery room, which minimized recall bias.

However, several limitations should be acknowledged. First, attrition occurred between the initial enrolled cohort (*n* = 2022) and the observed cohort (*n* = 898). Comparison between the initially enrolled cohort and observed cohort indicated statistically significant difference in several socio-demographic and obstetric characteristics (Supplementary Tables S1, S2). These differences may introduce selection bias if the factors associated with study participation are related to exposure (initiation of breast feeding) and outcome (postpartum depression). Although the multivariable regression adjusted several of these variables, residual bias cannot be excluded, and the results should therefore be interpreted taking this into consideration. The other limitation of the study is we did not measure the antepartum depression among women which strongly correlates with breastfeeding and postpartum depression as well as maternal-neonatal bonding disorders that may delay the initiation of breastfeeding. Third, the study was an exploratory study to assess the obstetric and neonatal characteristics associated with postpartum depression, so the sample size was not based on the early initiation of breastfeeding as a primary exposure for depressive symptoms. Fourth limitation was women who had cesarean section were excluded from this analysis, which limits the generalizability among women who have had vaginal birth in Nepal. Fifth limitation was the present analysis should be interpreted as exploratory since the parent cohort was not specifically designed to assess early initiation of breastfeeding as a primary exposure for postpartum depressive symptoms. Finally, the odds ratio can be overestimated when outcomes are common and since the study has high prevalence of postpartum depression, the reported estimates should not be interpreted as relative risk.

## Conclusion

This multi-centric cohort found that the delayed initiation of breastfeeding is associated with higher odds of postpartum depression. The association remained significant after adjusting to several socio-demographic and obstetric factors. These results suggest that supporting early initiation of breastfeeding benefits not only infants but also maternal psychological wellbeing. However, given the observation nature of the study, the result should be interpreted with caution. To minimize the long-term impact of maternal depression, it is essential to focus on breastfeeding within one hour of childbirth and provide proper counseling services for exclusive breastfeeding.

## Data Availability

The original contributions presented in the study are included in the article/Supplementary Material, further inquiries can be directed to the corresponding author.
