# Supplementary material for: Association of early initiation of breastfeeding on postpartum depression—multi-centric longitudinal cohort study in Nepal
Source: Front Glob Womens Health. 2026 May 15;7:1752660. doi: 10.3389/fgwh.2026.1752660 (PMC13219236; doi:10.3389/fgwh.2026.1752660)
Supplement: Supplementary file 1 [file Datasheet2.pdf]

06 March 2026

Comment 1. Selection process and representativeness

Although a study flow diagram has now been added, substantial attrition occurred between initial sampling (n=2022), observed participants (n=898), and those followed to 90 days (n=801). The supplementary tables indicate statistically significant differences between observed and non-observed groups across multiple socio-demographic and obstetric variables. However, the manuscript and response letter state that there were "no differences." This inconsistency must be corrected.

Response- We have now provided point to point response for comment 1.

Comment 1.1. Correct the description of group comparability.

Response. Correction of group comparability- We have revised the manuscript in lines 184-187 to clarify that statistically significant differences existed between the initial sampled population (n=2022) and the observed participants (n=898) in several socio-demographic and obstetric characteristics, including maternal education, ethnicity, parity, mode of delivery, preterm birth, and low birth weight (Supplementary Table 1). The previous statement indicating that there were "no differences" has been removed.

Comment 1.2. Clarify at which stage refusals/losses occurred

Response. Clarification of attrition /loss to follow up- The Methods and Results sections have been revised to clarify the stages of participant inclusion and loss on the initial sampling, clinical observation and follow-up in lines 96-105. We have mentioned the initial sampling of 2022 women were randomly selected from 21,805 eligible deliveries. The clinical observation was done among 898 women-infant had complete observation and follow up was done among 801 women-infant pair.

Comment 1.3. Provide a transparent description of how the 898 observed participants differ from the original 2022 sample

Response. Description of differences between the 2022 and 898 samples-We have added a description explaining that women included in the observed sample differed from the original sample cohort in several characteristics, as presented in supplementary Table 1 in the result section lines 184-187. Also, we have described it in the discussion section as one of the limitations in lines 284-290.

Comment 1.4. Revise the manuscript text to accurately reflect any observed imbalances

Response. The relevant sentence on difference and imbalance in the population characteristics between the original sample cohort and observed cohort has been revised in the methodological Considerations section in lines 284-290.

Comment 1.5. Explicitly discuss the potential for selection bias and its implications for internal and external validity

Response. Selection bias discussion- We have expanded the discussion section to address potential selection bias and its implications for internal and external validity in discussion section lines 287-289 and in the conclusion statement in lines 305-306.

Comment 1.6. If weighting, adjustment, or sensitivity analyses were performed to address this issue, these should be described

Response. Adjustment for imbalance-The population characteristics which were differing between the initial sample cohort and observed sample cohorts were maternal education, ethnicity, parity, preterm birth and birth weight. Some of these characteristics were adjusted in the multivariable regression in table 2. This has now been clarified in lines 289-291.

Comment 2. Sample size justification- There are contradictory statements regarding whether the study was powered to assess the association between early initiation of breastfeeding and postpartum depression.

We have now clarified it in the following sections.

Comment 2.1. Provide a transparent sample size calculation, including assumed effect size, exposure prevalence, outcome prevalence, alpha, and power.

Response. The sample size calculation has been clarified in the Methods section.

The original cohort was designed for broader maternal and neonatal outcomes. Based on previous studies in Nepal reporting approximately 21% prevalence of postpartum depressive symptoms and assuming a prevalence of delayed initiation of breast feeding of approximately 31%, we estimated that approximately 900 participants would provide 80% statistical power at a 5% significance level to detect an association between delayed breastfeeding initiation and postpartum depressive symptoms. We have explained this in lines 108-112.

Comment 2.2. Clarify whether the study was prospectively powered for this exposure–outcome relationship or whether the analysis is exploratory.

The original the cohort was not designed to evaluate early initiation of breast feeding as the primary exposure to postpartum depression and therefore, this is an exploratory analysis. This is provided in lines 112-114.

Comment 2.3. Ensure consistency between the Methods and Discussion sections.

Response- We have now added in the method section in lines 112-114 and in discussion section in lines 298-300 as “the present analysis should be interpreted as exploratory since the parent cohort was not specifically designed to assess early initiation of breastfeeding as a primary exposure for postpartum depressive symptoms”

Comment 3. Effect measure in a cohort design

The primary analyses rely on odds ratios despite the outcome prevalence being relatively high (~21–31%). In such contexts, odds ratios can materially overestimate relative risks.

Please:

i. Justify the use of odds ratios more rigorously, or
ii. Reanalyse the primary association using risk ratios (e.g., log-binomial or Poisson regression with robust variance).
iii. If odds ratios are retained, their interpretation must be appropriately qualified.

Response- We thank the you for this important methodological observation.

Odds ratio provides the ratio of outcome among exposed and outcome among non-exposed population, while relative risk is the relative ratio between outcome among exposed versus the outcome among total population cohort. This study was primed to explore what is ratio of the outcome (postpartum depression) among the exposed (early initiation of breast feeding) versus outcome among the non-exposed, so we used odds ratio.

The second reason is postpartum depressive symptoms in our study population had a prevalence of 21.2% overall, with 31.4% among women with delayed breastfeeding initiation. We acknowledge that odds ratios may overestimate the relative risk when outcomes are common. Logistic regression was initially used due to its robustness and comparability with previous literature examining postpartum depression.

We clarified the interpretation of odds ratios in the manuscript. The method section explicitly why odds ratios were used and not be interpreted as risk ratios in lines 167-170. Also, we have made it clear in the abstract lines 37-38 and main text conclusion in lines 302-303.

Comment 4. Role of SOC (mediator vs. confounder)

The DAG identifies SOC as a mediator. However, SOC is included in adjusted regression models without clear distinction between total and direct effects.

Please:

i. Clarify whether the primary objective is estimation of total or controlled direct effects.
ii. Align the modelling strategy with the causal framework.
iii. Clearly label models accordingly.

We thank you for highlighting this important issue regarding causal interpretation.

In our conceptual framework, **sense of coherence (SOC)** was considered a **potential mediator** between early initiation of breastfeeding and postpartum depressive symptoms.

To better align the analytical approach with the causal framework, we clarified the interpretation of the regression models.

- **Model I:** Unadjusted association between breastfeeding initiation and depressive symptoms
- **Model II:** Adjusted for confounders identified in the DAG (maternal education, age, ethnicity, parity, birth weight, mode of birth, infant sex) – representing the **total effect**
- **Model III:** Additionally adjusted for SOC – representing the **controlled direct effect**

These model interpretations have now been explicitly described in the Methods section in lines 158-165 and lines 173-176.

Comment 5. Internal consistency-Please reconcile discrepancies in reported prevalence of postpartum depressive symptoms across sections of the manuscript.

Response- Among the 801 women included in the analytical sample, 170 reported postpartum depressive symptoms at 90 days postpartum, corresponding to a prevalence of 21.2%. Among women with delayed initiation of breastfeeding, 31.4% reported depressive symptoms. We have mentioned that in lines 196-197.
