# Supplementary material for: Association of early initiation of breastfeeding on postpartum depression—multi-centric longitudinal cohort study in Nepal
Source: Front Glob Womens Health. 2026 May 15;7:1752660. doi: 10.3389/fgwh.2026.1752660 (PMC13219236; doi:10.3389/fgwh.2026.1752660)
Supplement: Supplementary file 3 [file Datasheet4.pdf]

27 March 2026

Comment 1. As the outcome is relatively common, odds ratios may overestimate the magnitude of association compared to relative risks. Please include a brief statement in the Methods or Discussion noting that odds ratios may overestimate effect sizes when outcomes are common, and the reported estimates should not be interpreted as relative risks.

Response- We have now added a brief statement in discussion section in lines 301-303, “the odds ratio can be overestimated when outcomes are common and since the study has high prevalence of postpartum depression, the reported estimates should not be interpreted as relative risk.”

Comment 2. Please soften the phrasing 'all possible confounders' as this is not strictly defensible in an observational study.

Response- We have revised the phrase “all possible confounders” as “possible confounders” in an observational study in line 211.
