# Supplementary material for: Association of early initiation of breastfeeding on postpartum depression—multi-centric longitudinal cohort study in Nepal
Source: Front Glob Womens Health. 2026 May 15;7:1752660. doi: 10.3389/fgwh.2026.1752660 (PMC13219236; doi:10.3389/fgwh.2026.1752660)
Supplement: Supplementary file 5 [file Table1.docx]

Supplementary table 1. Distribution of the demographic and obstetric characteristics in the 2022 population.

|  | Breastfeeding within 1 hour | |  |  |
| --- | --- | --- | --- | --- |
|  | No (427, 21.1%) | Yes (471, 23.3%) | Not observed  (1,124, 55.6%) | |
| Maternal education |  |  |  | <0.0001 |
| Educated (1296) | 254 (12.6%) | 305 (15.1%) | 737 (36.5%) |  |
| Uneducated (178) | 61 (3.0%) | 37 (1.8%) | 80 (4.0%) |  |
| Not available (548) | 112 (5.5%) | 129 (6.4%) | 307 (15.2%) |  |
| Maternal age |  |  |  | 0.136 |
| <18 (80) | 19 (0.9%) | 21 (1.0%) | 40 (2.0%) |  |
| 19-24 (1017) | 219 (10.8%) | 244 (12.0%) | 554 (27.4%) |  |
| 25-29 (639) | 139 (6.9%) | 144 (7.1%) | 356 (17.6%) |  |
| 30-34 (224) | 43 (2.1%) | 41 (2.0%) | 140 (6.9%) |  |
| ≥35 (62) | 7 (0.35%) | 21 (1.0%) | 34 (1.7%) |  |
| Ethnicity |  |  |  | <0.0001 |
| Advantaged (674) | 95 (4.7%) | 160 (7.9%) | 419 (20.7%) |  |
| Disadvantaged (1348) | 332 (16.4%) | 311 (15.4%) | 705 (34.9%) |  |
| Parity |  |  |  | 0.001 |
| No previous birth (790) | 134 (6.6%) | 183 (9.1%) | 473 (23.4%) |  |
| 1 previous birth (503) | 107 (5.3%) | 119 (5.9%) | 277 (13.7%) |  |
| ≥2 previous birth (729) | 186 (9.2%) | 169 (8.4%) | 374 (18.5%) |  |
| Mode of delivery |  |  |  | <0.0001 |
| Spontaneous vaginal (1457) | 404 (20.0%) | 457 (22.6%) | 596 (29.5%) |  |
| Assisted (60) | 21 (1.0%) | 14 (0.7%) | 25 (1.2%) |  |
| Non-responder (505) | 2 (0.1%) | 0 (0.0%) | 503 (24.9%) |  |
| Preterm |  |  |  | <0.0001 |
| No (1942) | 417 (20.6%) | 438 (21.7%) | 1087 (53.8%) | |
| Yes (80) | 10 (0.5%) | 33 (1.6%) | 37 (1.8%) |  |
| Low Birth Weight |  |  |  | 0.012 |
| No (1571) | 321 (15.9%) | 389 (19.2%) | 861 (42.6%) |  |
| Yes (451) | 106 (5.2%) | 82 (4.1%) | 263 (13.0%) |  |
| Infant's sex |  |  |  | 0.954 |
| Girl (905) | 194 (9.6%) | 210 (10.4%) | 501 (24.8%) |  |
| Boy (1116) | 233 (11.5%) | 261 (12.9%) | 622 (30.8%) |  |
| SOC at 90 days |  |  |  | 0.014 |
| High (619) | 118 (5.8%) | 169 (8.4%) | 332 (16.4%) |  |
| Low (1403) | 309 (15.3%) | 302 (14.9%) | 792 (39.2%) |  |
