# Supplementary material for: Association of early initiation of breastfeeding on postpartum depression—multi-centric longitudinal cohort study in Nepal
Source: Front Glob Womens Health. 2026 May 15;7:1752660. doi: 10.3389/fgwh.2026.1752660 (PMC13219236; doi:10.3389/fgwh.2026.1752660)
Supplement: Supplementary file 6 [file Table2.docx]

Supplementary table 2. Distribution of the demographic and obstetric characteristic in 898 population

|  | Yes (471, 52.5%) | No (427, 47.6%) |  |
| --- | --- | --- | --- |
| Maternal education |  |  | 0.008 |
| Educated (559) | 305 (34.0%) | 254 (28.3%) |  |
| Uneducated (98) | 37 (4.1%) | 61 (6.8%) |  |
| Non-responder (241) | 129 (14.4%) | 112 (12.5%) |  |
| Ethnicity |  |  | <0.0001 |
| Advantaged (255) | 160 (17.8%) | 95 (10.6%) |  |
| Disadvantaged (643) | 311 (34.6%) | 332 (37.0%) |  |
| Maternal age | Yes | No | 0.168 |
| <18 (40) | 21 (2.3%) | 19 (2.1%) |  |
| 19-24 (463) | 244 (27.2%) | 219 (24.4%) |  |
| 25-29 (283) | 144 (16.0%) | 139 (15.5%) |  |
| 30-34 (84) | 41 (4.6%) | 43 (4.8%) |  |
| ≥35 (28) | 21 (2.3%) | 7 (0.8%) |  |
| Parity |  |  | 0.032 |
| No previous birth (317) | 183 (20.4%) | 134 (14.9%) |  |
| One previous birth (226) | 119 (13.3%) | 107 (11.9%) |  |
| More than one previous birth (355) | 169 (18.8%) | 186 (20.7%) |  |
| Mode of birth |  |  | 0.104 |
| Spontaneous vaginal (861) | 457 (50.9%) | 404 (45.0%) |  |
| Assisted Vaginal (35) | 14 (1.6%) | 21 (2.3%) |  |
| Non-responder (2) | 0 (0.0%) | 2 (0.2%) |  |
| Preterm birth |  |  | 0.001 |
| No (855) | 438 (48.8%) | 417 (46.4%) |  |
| Yes (43) | 33 (3.8%) | 10 (1.1%) |  |
| Low Birth weight |  |  | 0.006 |
| No (710) | 389 (43.3%) | 321 (35.8%) |  |
| Yes (188) | 82 (9.1%) | 106 (11.8%) |  |
| Sex |  |  | 0.799 |
| Girl (404) | 210 (23.4%) | 194 (21.6%) |  |
| Boy (494) | 261 (29.1%) | 233 (26.0%) |  |
| SOC at 90n days |  |  | 0.008 |
| High SOC (287) | 169 (18.8%) | 118 (13.1%) |  |
| Low SOC (611) | 302 (33.6%) | 309 (34.4%) |  |
